# Nutrient and Antinutrient Compositions and Heavy Metal Uptake and Accumulation in* S. nigrum* Cultivated on Different Soil Types

**DOI:** 10.1155/2018/5703929

**Published:** 2018-01-18

**Authors:** Adijat Funke Ogundola, Callistus Bvenura, Anthony Jide Afolayan

**Affiliations:** Medicinal Plants and Economic Development (MPED) Research Centre, University of Fort Hare, Private Bag X1314, Alice 5700, South Africa

## Abstract

*Solanum nigrum *cultivated on different soil texture types, sandy clay loam, silty clay loam, clay loam, loam, and control soils, were evaluated for proximate compositions, antinutrients, vitamins, and mineral composition with plant age using standard analytical methods. Accumulation of trace elements using translocation factor was studied to determine their toxic levels in plant tissues. Data were analysed by ANOVA and results expressed as means and standard deviation. Ash content, crude fibre, protein, alkaloid, phytate, and saponin ranged between 11.4 and 12%, 19.24 and 19.95%, 34.23 and 38.98, 42.08 and 45.76 mg/ml, 0.84 and 1.17%, and 94.10 and 97.00%, respectively. Vitamins A, C, and B were present in high quantity. Macro- and micronutrients recorded showed that* S. nigrum *is a potential reservoir of minerals. Accumulation of micronutrients was observed to be the highest at the flowering stage between the 4th and 5th weeks after transplanting. Plants cultivated on clay loam, silty clay loam, and loam soils accumulated elevated nutritional compositions and abundant antinutrients. However, the accumulated trace metals in the plants are within the recommended safe levels. All nutrient values are in the recommended requirements for daily consumption.

## 1. Introduction

Today, consumers are constantly searching for plant based foods with nutritional and pharmacological benefits. Moreover, there has been a recent upsurge in the use of wild plants as sources of food and healing properties. It is therefore not surprising that an estimated 1 billion people in the world use wild plants [1]. The consumption of food that is low in nutritional compositions has been traced to the global burden of diseases. Besides their nutritional role, wild vegetables are also proving to be promising natural antioxidant source [2, 3]. The nutritional role of wild vegetables in the diet has been receiving renewed attention and has been widely reported [4, 5]. Also, the dependence of rural dwellers on wild vegetables for food among other uses has been revealed through numerous reports [6, 7]. Wild vegetables provide a cheap and affordable source of some important micronutrients that are needed to complete a balanced diet [8, 9]. The renewed interests in wild vegetables have also created awareness among the people on their numerous benefits and are slowly becoming targets for nutrient-intervention programmes in South Africa.

However, it becomes so important to consider that the intake of excess micronutrients may be toxic to health [10]. Micronutrients, unlike degradable organic chemicals, have attributes of metals that are difficult to eliminate by metabolic degradation if they bioaccumulate in the tissues [11]. Some are also reported to be toxic at low concentrations [12–14]. Concentrations of mineral nutrients in plants fluctuate greatly in space and time due to environmental factors such as weather, climate, and soil physicochemical properties [15, 16]. Some authors have reported variations in mineral uptake in plants due to soil compositions [17, 18]. The ability of some mineral nutrients to become toxic at some concentrations warrants the checking of minimum toxicity levels of consumption. Hazard evaluation strategies for their bioaccumulation are very essential [8, 19]. Moreover, the ingestion of these toxic minerals beyond safe levels normally causes human organ or tissue damage [20].

Among the notable and important wild vegetable species in Africa is* Solanum nigrum *[21]. Its nutritional value has been extensively investigated by different researchers and recommended as an immune booster [22–26]. However, there is a dearth of information on the influence of soil texture types on this plant's nutrient compositions and the trend of accumulation of metals at various stages of growth.

The nutritional compositions of* S. nigrum* could be influenced by soil texture modulation at various growth stages [17, 27]. Sinclair [28] also reported that the growth performance of plants is positively related to the available minerals in the soil. To corroborate this report, Van Averbeke et al. [29] showed the need for the availability of sufficient nitrogen in the soil in order to achieve optimum growth in some* Solanum* species. In addition, the nutrient and micronutrient composition in* S. nigrum* at every stage of development is necessary to be investigated especially when cultivated on different soil types. This information is conceived to assist in ascertaining the best possible time for harvesting the leaves for maximal potential. Soils of different texture types are a physical condition, with different water holding capacity, nutrient compositions, aeration, drainage, and friability [30]. All these parameters may possibly influence the levels of composition of nutrients, antinutrients, vitamins, and trace metals in plants. This study based its investigation on the influence of soil texture types on availability of all mentioned parameters in* S. nigrum*. Within this framework, the study investigated mineral compositions in* S. nigrum* as influenced by different soil textures at different growth stages. Also, toxicity levels of different micronutrients in the plants were investigated to ascertain their ability to be absorbed and the levels of absorption.

## 2. Materials and Methods

### 2.1. Study Site, Soil Collection, and Preparation

Experimental soil was collected from a fallow land at a depth of 30 cm from the University of Fort Hare farm, Alice Campus, South Africa. The farm was located at 32°46′47′′S and 26°50′5′′E and 524 m a.s.l. The soil was air-dried, ground, sieved, and separated into sand, silt, and clay particles; soil types were formulated by relative combinations of sand, silt, and clay in different ratios as follows: sandy clay loam (ST_1_): 60, 30, and 10%; silty clay loam (ST_2_): 10, 60, and 30%; clay loam (ST_3_): 36, 30, and 34%; loam (ST_4_): 40, 40, and 20%; and the control soil as shown in Table 1. Soil formulation followed the triangular soil classification system of SSDS [31] and USDA [32]. Three soil samples were taken from each of the soil types and analysed for their physicochemical properties as displayed in Tables 2(a) and 2(b) for the two trials, respectively.

### 2.2. Plant Materials and Planting

*Solanum nigrum* was initially collected in the wild in Alice, identified, and deposited in the Giffen herbarium of the university with voucher number (BVE11/017) [33]. Seeds were extracted from matured and ripe berries, washed in distilled water, and dried at room temperature on the laboratory work bench for 2 days and stored in sealed bottles. Seedlings were raised in nursery trays in the glass house and a single seedling was transplanted at four-leaf stage into each experimental pot measuring 25 cm in diameter, containing 5 kg of soil. The experiment was conducted twice on 4 October 2015 and 6 February 2016 for the first and second trials, respectively. Agronomic practices included watering of the seedlings twice daily. Foliar application of Multifeed Water Soluble Fertiliser 43 (3 g/L) commenced in the 2nd week after transplanting (ATP) and was applied at the same rate, once per week in both trials.

### 2.3. Experimental Design, Plant Processing, and Analysis

Each trial was organised in a Randomised Complete Block Design (RCBD) with three replicates. Plants were harvested from the different soil treatments every week. This commenced from the first week till the sixth week after transplanting (ATP). Dried shoots were pulverised using an electric motor blender and kept in an airtight glassware container and stored at 4°C until when needed. With the exception of minerals, samples required for proximate and vitamins A, C, and E analysis were harvested at 4 weeks (ATP) and processed. The pulverised samples were used for analyses of all the nutritive and antinutritive compositions as well as vitamins A, C, and E. However, vitamin A was evaluated from fresh plants and all the analyses were carried out in triplicate. The concentrations of Ca, K, Mg, Na, P, Cu, Mn, and Zn in the soil were determined using the Inductively Coupled Plasma-Optical Emission Spectrometer (ICP-OES) using standard methods as outlined by AGRILASA [34].

### 2.4. Effect of Soil Types on Proximate Composition

Moisture content was determined as described by AGRILASA [34]. Empty labelled porcelain crucibles were oven dried at 105°C for one hour, cooled in a desiccator, and weighed (*W*_1_). Pulverised samples of* S. nigrum* from soil treatments each weighing 1 g ± 0.001 (*W*_2_) were put into the crucibles and oven dried at 105°C to constant weight. This was then put in a desiccator to cool, after which it was weighed (*W*_3_). The percentage moisture content was calculated as

(1) $$\begin{array}{c} \text{\% Moisture content}=\frac{W_{2}-W_{3}}{W_{2}-W_{1}}\times 100. \end{array}$$

### 2.5. Determination of Ash Content

The method of AGRILASA [34] was adopted in determining the ash content. Porcelain crucibles were labelled with a heat resistant marker and dried at 105°C for 1 hour, left to cool in a desiccator, and weighed (*W*_1_). About 1 g of the pulverised samples from different soil treatments was placed in the preweighed porcelain crucibles (*W*_1_) and reweighed (*W*_2_) in order to determine the weight loss. The labelled crucibles with the contents were arranged in the muffle furnace programmed to ash initially at 250°C for 1 hour and consequently at 550°C for 5 hours. The crucibles containing the ash were removed after complete ashing, cooled in a desiccator, and weighed (*W*_3_). The percentage ash was calculated as

(2) $$\begin{array}{c} \%\text{ Ash content}=\frac{W_{2}-W_{3}}{W_{2}-W_{1}}\times 100. \end{array}$$

### 2.6. Determination of Crude Fibre

This analysis employed the modified method described by Antia et al. [35]. About 2 g of pulverised samples of* S. nigrum* was digested separately in Kjeldahl flasks by boiling in 100 ml of 1.25% of concentrated H_2_SO_4_ and a digestion tablet (catalyst) until the mixture was clear. The residue was rinsed with boiling water several times to remove the content totally. The residue was also rinsed with 100 ml of 1.25% NaOH solution. The final residue was then dried at 100°C, cooled in a desiccator, and weighed (*C*_1_). The samples were then incinerated in a muffle furnace at 550°C for 4 h, then transferred to cool in a desiccator, and reweighed (*C*_2_). The percentage crude fibre was calculated as

(3) $$\begin{array}{c} \%\text{ Crude fibre}=\frac{C_{1}-C_{2}}{\text{Weight of original sample}}\times 100. \end{array}$$

### 2.7. Determination of Crude Fats (Lipid)

About 1 g of pulverised of* S. nigrum* was weighed into preweighed flasks and extracted in 100 ml of diethyl ether and then placed on an orbital shaker for 24 h. The mix was then filtered and the ether extracts were collected differently in previously weighed (*W*_1_) clean beakers. It was thereafter equilibrated with diethyl ether to 100 ml and shaken for another 24 h and the filtrate was collected in the same beaker (*W*_1_). The ether was concentrated to dryness in a steam bath and dried in an oven at 40–60°C for complete dryness and the beaker was reweighed (*W*_2_). The crude fat content was calculated as

(4) $$\begin{array}{c} \%\text{ Crude fat}=\frac{W_{2}-W_{1}}{\text{Weight of original sample}}\times 100. \end{array}$$

### 2.8. Determination of Crude Protein

Pulverised samples 2 g of each soil treatment of* S. nigrum* were digested in a Kjeldahl flask by boiling in 20 ml of concentrated H_2_SO_4_ and a digestion tablet (catalyst) until the mixture was clear. The digest was filtered and marked up to 250 ml and then distilled. The aliquot (50 ml of 45% sodium hydroxide solution) was transferred into a 500 ml round bottom flask and distilled. 150 ml of the distillate was collected into a flask containing 100 ml 0.1 N HCl. This was then titrated against 2.0 M NaOH using methyl orange as indicator to give a colour change of yellow (end point). The % nitrogen content was calculated as

(5) $$\begin{array}{c} \frac{\left[\left(\text{ml standard acid}\times N\text{ of acid}\right)-\left(\text{ml blank}\times N\text{ of base}\right)\right]-\left(\text{ml std base}\times N\text{ of base}\right)\times 1.4007}{\text{Weight of sample in grams}}, \end{array}$$

where *N* is normality.

Percentage crude protein was obtained by multiplying the nitrogen value by constant factor (6.25). % crude protein is nitrogen in sample × 6.25.

### 2.9. Determination of Carbohydrate

The carbohydrate content was calculated by subtracting the total crude protein, crude fibre, ash, and lipid from the total dry matter as

(6) %  Total Carbohydrate=100−%  Moisture content+%  Total Ash+%  crude Fat+%  Crude Fibre+%  Crude Protein.

### 2.10. Energy Content

For each of the* S. nigrum* treatments, the kilocalorie (Kcal/100 g) value estimation was done by summing up the multiplied values for crude protein, crude lipid (excluding crude fibre), and carbohydrate, using the factors (4 kcal, 9 kcal, and 4 kcal), respectively. Energy content was thus calculated as

(7) Energy value kcal/100 g=crude protein×4+crude fat×9+total carbohydrate×4.

### 2.11. Determination of Vitamins

For all the vitamins, the method of Pearson [36] was employed.

*Vitamin A*. 1 g of pulverised samples of* S. nigrum *from different soil types was macerated with 20 ml of petroleum ether. This was decanted into a test tube and then evaporated to dryness. 0.2 ml of chloroform-acetic anhydride (1 : 1, v/v) was added to the residue. About 2 ml of TCA-chloroform (1 : 1 v/v) was added to the resulting solution and absorbance was measured at 620 nm. The vitamin A standard was prepared in like manner and the absorbance was taken at 620 nm. The concentration of vitamin A in the sample was extrapolated from the standard curve.

*Vitamin C*. About 1 g of pulverised samples of* S. nigrum *from different soil types was macerated with 20 ml of 0.4% oxalic acid. This was filtered and to 1 ml of filtrate 9 ml of indophenol reagent was added. The standard solution of vitamin C was prepared similarly and the absorbance of the standard solution and the sample was read at 520 nm. The concentration of vitamin C was extrapolated from the standard curve of vitamin C.

*Vitamin E*. About 1 g of the pulverised samples of* S. nigrum *from different soil types was macerated separately with 20 ml of ethanol and then filtered. A mixture of 0.2% ferric chloride in ethanol and 1 ml of 0.5%  *α*-*α*-dipyridine was added to 1 ml of the filtrate. This was distilled to 5 ml. Absorbance was taken at 520 nm. The standard solutions were prepared similarly and the concentration of vitamin E was extrapolated from the standard curve.

### 2.12. Effect of Soil Types of Antinutritive Composition

The modified titration method of Sanchez-Alonso and Lachica [37] was employed to determine the oxalate content of* S. nigrum*. The oxalate content was calculated by taking 1 ml of 0.05 M of KMnO_4_ equivalent to 2.2 mg oxalate which was multiplied by the titre value of each of the plant treatments as follows: oxalate content = titre value × 2.2 mg.

Phytic acid was determined by the method described by Olayeye et al. [38]. Phytic acid was calculated in % as follows: (titre value × 0.00195 × 1.19 × 100) g.

Saponin content was determined as described by Obadoni and Ochuko [39]. The saponin content was calculated using the following equation:

(8) %  Saponin content=weight of residueweight of original sample×100.

Alkaloid content of different samples of* S. nigrum *was determined according to the method of Omoruyi et al. [40].

The alkaloid content was calculated as

(9) $$\begin{array}{c} \%\text{ alkaloid}=\frac{\text{weight of precipitate}}{\text{weight of original sample}}\times 100. \end{array}$$

### 2.13. Effect of Soil Types on Macro- and Microminerals Uptake in* S. nigrum*

The method described by Bvenura and Afolayan [41] was used for the digestion of plant material. The tubes were allowed to cool and three successive portions of 1 ml hydrogen peroxide were added at 10 s intervals due to the volatility of the reaction. The tubes were returned to the block digester at a temperature of 330°C and were removed from the block digester when the digest turned clear in colour. The tubes were allowed to cool to room temperature, contents were transferred into 50 ml volumetric flasks, and then deionized water was added to attain volumes of 50 ml. Standards were prepared for all the individual elements to be analysed. The macrominerals (calcium, magnesium, potassium, sodium, and phosphorus) and microminerals (iron, zinc, aluminium, manganese, and copper) were determined using the Inductively Coupled Plasma-Optical Emission Spectrometer (ICP-OES; Varian 710–ES series, SMM Instruments, Cape Town, South Africa). All analyses were carried out in triplicate.

### 2.14. Effect of Soil Types on Phytoextraction Level of Trace Elements (Heavy Metals) in* S. nigrum*

This was determined by evaluating the quantity of each of the micronutrient/heavy metals (Fe, Al, Zn, Cu, and Mn) in root/shoot ratios as follows:

TF (translocation factor) given as [metals] shoot/[metals] root [42, 43].

## 3. Statistical Analysis

All experiments were done in triplicate and the results were expressed as mean ± SD using the Microsoft excel 2010 spreadsheet. Data were subjected to statistical analysis using MINITAB Release 17. A one-way analysis of variance (ANOVA) was used to compare the values of proximates, nutrients, antinutrients, and trace element levels accumulated by plants among different soil treatments. Means were segregated using Fisher's Least Significant Difference (LSD) pairwise comparison. The means were treated as significantly different at *p* < 0.05.

## 4. Results

The physical properties and particle compositions of the soil treatments used for the cultivation of* S. nigrum* are shown in Table 1. The physicochemical analytical results of soils before the first and second trials are presented as Tables 2(a) and 2(b).

### 4.1. The Effect of Soil Types on Proximate Composition in* S. nigrum*

In this present study, significant variations were found on* S. nigrum* cultivated on different soil texture types with respect to their proximate compositions such as crude fibre, ash, moisture, crude protein, lipid, carbohydrate, and energy value (Table 3(a)). The crude fibre values ranged from 19.24 to 19.95% in the first trial and 18.07 to 19.95% in the second trial (Table 3(a)). However, the highest values were recorded in* S. nigrum *from clay loam soils in both trials, while the minimum fibre was recorded in plant samples from control soils. Ash contents in the first trial were the highest (12.42%) in* S. nigrum* samples from clay loam soils and 11.54% from silty clay loam in the second trial. Likewise the highest moisture contents (96.05%, 94.64%) were recorded in* S. nigrum *samples from clay loam and silty clay loam soils in the first and second trials, respectively. However, the highest protein 38.98% was recorded in samples from clay loam soils in the first trial and 37% in loam soils in the second trial (Table 3(a)). Of all soil treatments, control soils recorded the lowest values in all the proximate parameters in both trials (Table 3(a)).

Significant differences were recorded in lipid, carbohydrate, and energy values of the plant samples from different soil treatments (Table 3(b)). The values ranged from 3.57 to 4.86% and 3.46 to 4.42% in the first and second trials, respectively. The lowest lipid values 3.57 and 3.46% were recorded in plant samples from clay loam soils in first and second trials, respectively. However, carbohydrate was the highest (16.85 and 17.99%) in samples from sandy clay loam and the control soils in the first and second trials, respectively. The lowest values (12.74 and 14.17%) were recorded in clay loam soils in the first and second trials, respectively. Samples from the loam and clay loam soils recorded the highest energy values (205.79 and 195.89 Kcal/100 g) in the first and second trials, respectively. However, samples from control soils had the lowest values (Table 3(b)).

### 4.2. Effect of Soil Types on Vitamins A, C, and E in* S. nigrum*

For vitamin A, values ranged from 117 to 137 mg/kg in* S. nigrum* from different soil types in the two trials. The highest vitamin A was recorded in plant samples on loam soils in both trials, while the lowest was noted in control soil plant samples. The highest value of vitamin A was noted in samples on loam soils. In both trials, the highest vitamin C was recorded in plant on loam soils and clay loam soils, while the lowest was noted in control soils. Vitamin E followed the same trend with vitamin A in both trials. There were no significant differences in vitamin A values among samples on silty clay loam, clay loam, and loam soils. However, the highest vitamin A values were observed in plants on loam soils in both trials. The highest vitamin E was recorded in plant samples on loam soils but there was no significant difference from plant samples on silty clay loam and loam soil samples in the first trial. However, there was significant difference among the different samples in the second trial (Table 3(c)). In this study, all the vitamins values in* S. nigrum *on both loam and clay loam are not significantly different except in the second trial of vitamin E. The highest values for all vitamins in both trials are recorded in loam soils and the lowest in control soils. The order of decrease in values is ST_4_> ST_3_ > ST_2_ > ST_1_ > ST_0_ in both trials for vitamins A and E, while in vitamin C it is ST_3_ = ST_4_ > ST_2_ > ST_1_ > ST_0_ (Tables 3(a), 3(b), and 3(c), resp.).

### 4.3. Effect of Soil Types on Antinutrient Composition in* S. nigrum*

Antinutrients in* S. nigrum *were significantly varied with different soil treatments (Table 4). The phytate content in* S. nigrum *ranges from 0.84 to 1.17 mg/kg and 0.88 to 1.15 mg/kg in the first and second trials, respectively. Highest 1.17 and 1.15 mg/kg were recorded in plants grown in clay loam soils in trials 1 and 2, respectively. The lowest 0.84 and 0.88 mg/kg were recorded in control soils in trials 1 and 2, respectively. Oxalate content of* S. nigrum *ranged from 11.00 to 15.25% with the highest (15.25 and 14.60%) in clay loam soils in trials 1 and 2, respectively. The lowest was observed in the control soils in trials 1 and 2, respectively. Saponin content was significantly high (97.00% and 97.80%) in sandy clay loam and control soils plant samples in the first and second trials, respectively. However, the lowest values (94.10 and 94.17%) in both trials were recorded in plant on clay loam soils. The highest alkaloids (45.78% and 44.10%) were recorded in plants on silty clay loam and clay loam soils in the first and second trials, respectively. The lowest values (43.03 and 42.08%) were recorded in plant samples from control soils as shown in Table 4.

### 4.4. Effect of Soil Types on Macronutrient Uptake in* S. nigrum*

Generally, in the two trials of the present study, the mineral nutrients (nitrogen, phosphorus, potassium, calcium sodium, and magnesium) in* S. nigrum *shoots were significantly different among soil treatments. The results exponentially increased from the transplanting stage to the 3rd week after transplanting (ATP). The values either stabilised or reduced between the 5th and 6th weeks ATP. The plant samples on clay loam or silty clay loam soils recorded the highest results and the lowest result was majorly recorded in the plant samples on control or sandy clay soils. The results varied in the two trials (Table 5(a)(i) (the first trial) and Table 5(e)(ii) (the second trial)).

*Solanum nigrum *samples on clay loam soils recorded the highest nitrogen values from the 2nd to the 6th weeks (ATP), while the lowest were recorded in control or sandy clay loam soils throughout the first trial. In the second trial, clay loam soil plant samples recorded the highest nitrogen value throughout the experiment and the control samples had the lowest values (Table 5(a)(i) (the first trial) and Table 5(a)(ii) (the second trial)), respectively.

The highest phosphorus value was observed in samples cultivated on silty clay loam soils in the 1st and 3rd weeks (ATP) but clay loam soil samples recorded the highest values in weeks 4 to 6 (ATP). In the second trial, the highest levels of phosphorus were recorded in samples from clay loam soil in the first and second weeks but samples on silty clay loam were elevated levels of phosphorus in the 3rd week. However, the highest phosphorus was noted in* S. nigrum *samples on clay loam soils from the 4th week till the 6th week ATP (Table 5(a)(i) (the first trial) and Table 5(a)(ii) (the second trial)), respectively.

The highest amount of calcium was noted in silty clay loam soils between the 1st and 2nd weeks (ATP) and clay loam soil recorded the highest values from the 3rd to the 6th weeks. The lowest calcium was recorded in the control soil treatment as shown in Table 5(b)(i) (the first trial) and Table 5(b)(ii) (the second trial). Potassium was the highest in* S. nigrum *cultivated on silty clay loam soil from the transplanting stage to the 2nd week, but samples raised on clay loam soil had the highest potassium from the 3rd week to 6th week in trials 1 and 2 (Table 5(b)(i) (the first trial) and Table 5(b)(ii) (the second trial)), respectively.

Sodium concentration exponentially increased from the time of transplanting to the 4th week and then began to drop at 6 weeks (ATP). The highest concentration was observed in* S. nigrum *samples from silty clay loam in the 1st week (ATP). Clay loam soils had the highest sodium content from week 2 to week 5 after transplanting. However, sandy clay loam plant samples accumulated the highest sodium value. The results of the two trials followed the same trend (Table 5(c)(i) (the first trial) and Table 5(c)(ii) (the second trial)). The highest amount of magnesium was recorded in* S. nigrum* samples cultivated on silty clay loam two weeks ATP.

### 4.5. Effect of Soil Types on Phytoextraction Level of Trace Elements (Heavy Metals) in* S. nigrum*

Measurable levels of the potentially toxic copper, zinc, iron, and manganese were present at variable levels in* S. nigrum* samples on all soil treatments. Difference was observed in the accumulation levels of all the micronutrients among* S. nigrum* samples of different soil treatments in the first and second trials (Tables 5(g)(i) and 5(g)(ii), resp.).

In the first trial, zinc accumulation was significantly high in* S. nigrum *samples cultivated on clay loam soils in the first week, while silty clay loam soil samples accumulated the highest from 2nd to 5th week after transplanting. However, there were no significant differences among* S. nigrum *samples on sandy clay loam and silty clay loam but the highest zinc accumulation was noticed on clay loam samples. Lowest zinc was observed in control soil plant samples in the first trial, while lowest was recorded in clay loam soil plant samples between first and second weeks ATP. However, significant high value (*p* < 0.01) was recorded in plant samples cultivated on silty clay loam soils from week 3 to week 6 ATP. The highest zinc value was observed in the 4th week in plant samples on the silty clay loam soils, in plant samples on sandy clay loam in the 5th week and samples on silty clay loam in the 6th week (Table 5(d)(i) (the first trial) and Table 5(d)(ii) (the second trial)).

Iron accumulation varied but the lowest amount was recorded from samples cultivated on the control soils. Silty clay loam soils recorded the highest iron values in the second week after transplanting but clay loam soil samples had the highest accumulation from weeks 3 to 6 after transplanting. The second trial followed the same trend as in the first trial (Table 5(d)(i) (the first trial) and Table 5(d)(ii) (the second trial)).

In the 1st and 2nd weeks of the first trial, there were no significant differences in copper accumulation among different samples. However, samples cultivated on silty clay loam soils had the highest amount of copper from weeks 3 to 6. In the second trial, there were no significant differences in the first week alone. Samples cultivated on silty clay loam soil recorded the highest values from weeks 3 to 6 except in week 2 when samples cultivated on clay loam soil recorded the highest values (Table 5(e)(i) (the first trial) and Table 5(e)(ii) (the second trial)).

Accumulation of manganese in the 1st week of the first trial showed no significant difference among plant samples on different soil. However, significant differences were recorded 2 to 6 weeks after transplanting. The highest amount of manganese was recorded in samples from silty clay loam soil in the 2nd and 4th–6th weeks and increased drastically in clay loam samples in the 3rd week of the first trial. The results in the second trial showed significant differences among plant samples on different soil types. However, plant samples on clay loam soils had the highest amount of manganese throughout the trial (Table 5(e)(i) (the first trial) and Table 5(e)(ii) (the second trial)).

### 4.6. Effect of Soil Types on Phytoextraction Level of Trace Elements (Heavy Metals) in* S. nigrum*

Generally in the current study, all micronutrients such as Fe, Zn, Mn, and Cu accumulated significant levels at different stages of plant growth on different soil types. The translocation factors increased as the plant advanced in age regardless of the soil types and the increase was the highest between 5th and 6th weeks after transplanting. At the 5th week when the highest concentrations of micronutrients were recorded, the translocation factor increased, stabilised, or reduced (Table 5(e)(i) (the first trial) and Table 5(f)(ii) (the second trial)).

## 5. Discussion

The proximate composition, antinutrients, macro- and microelement uptake, and compositions in* S. nigrum *shoots cultivated on different soils differed significantly in the present study. Variations were also observed in vitamins A, C, and E uptake on different soil types. Ash content is a measure of the nutritional value of food which is regarded as the reflection of the mineral contents preserved in food materials [44]. Usually ash content does not exceed 5%; however, in the present study,* S*.* nigrum* recorded high ash content ranging between 11.14 and 12.42% in all treatments and this corresponds to compositions found in processed foods [43]. High moisture at harvest (94–96.05%) recorded in* S. nigrum* vegetable in this study rendered it perishable and reduces the storage lifespan. It is usually accompanied with physiological changes and biochemical reactions that lead to microbial growth. More than 94% moisture content was recorded for all samples of* S. nigrum *from different soils in the current study. The importance of fibre in vegetables is its peculiar role at increasing glucose tolerance and insulin sensitivity [45]. In addition, its high contents in vegetables function as a serum cholesterol reducing agent and this prevents the risk of coronary heart disease and/or hypertension [46]. High fibre contents (19.24–19.95%) in both trials of the present study are in close range to 18.8% reported in* S. nigrum *by Afolayan and Jimoh [47]. The Recommended Daily Allowance (RDA) of 19–25, 21–38, 28, and 29 g, respectively, for children, adults, pregnant women, and lactating mothers, respectively, can conceivably be achieved in* S. nigrum *from all soil treatments in this study [25]. Protein values (34.23 to 38.98%) were higher than those reported in* Chenopodium album *and* Sonchus asper *[47]. These authors reported values ranging between 13.25 and 26.44% in their study. Dietary fats/lipids add to the palatability of food through absorption and retaining its flavour [44]. The lipid compositions (3.46–4.86%) in* S. nigrum *from the present study are less than 8.57% reported in the study of Afolayan and Jimoh [47]. However, the results are within the range reported by Akubugwo et al. [25] and Afolayan and Bvenura [48].* Solanum nigrum *in this study has not proved to be a very good source of high carbohydrate because of the lower content [49]. However, carbohydrate compositions (13.59–17.99%) recorded in the current study are higher than 5.1% that was earlier reported by Picciano [50]. The energy values (172.51 and 205.70%) recorded in control soils (lowest) and loam soils (highest), respectively, were higher than those recorded in other wild vegetables. In this study, the significant variations experienced in yield and quality of all chemical compositions accumulated in* S. nigrum *were conceivably due to physical, chemical, and biological properties of the soil on which the plants were cultivated [51].

The presence of vitamins and several mineral elements has been reported to improve the quality and dietary composition of vegetables [51]. Vitamin A functions in the body apart from helping in growth also promote resistance to disease, delay ageing, and preside over the health of the eyes, nails, and hairs [52]. Vitamin C helps in the health of lungs and bronchia, teeth and gums, and bones and joints and purifies the blood. It also prevents the scavenging activities of toxicants that trigger the inflammatory cascade and is associated with reduced severity of inflammatory conditions such as asthma, osteoarthritis, and rheumatoid arthritis [53, 54]. It could also be used in the herbal medicine for the treatment of common cold and prostate cancer [50]. Vitamin E has been reported as a good antioxidant necessary for the formation of red blood cells and the structure, recovery, and maintenance of muscle and other tissues [55]. The little quantity could even be supplemented with antioxidants which are present naturally in wild vegetables including* S. nigrum *and this assists in reducing the incidence of degenerative processes [56]. Vitamin C (ascorbic) is a common antioxidant present in both plant and animal cells [57]. It was abundantly present in these trials; however, it is the most common antioxidant present in both plant and animal cells and plays a major role in human health [57].

The values obtained in this study for all antinutrient compositions revealed low levels of phytates, oxalate, and moderate levels of alkaloids. However, the saponin content was high although these are believed to be significantly reduced during the process of cooking since they are heat labile [58]. The levels of phytate obtained in this study are comparable with previously reported values for the same vegetable [59]. However, these chemical compounds in plants mediate their effects on the human body through the same processes as conventional drugs thus enable the plant to work as effective as conventional medicines [60].

Mineral nutrients are indispensable due to their major roles in human die. The role of mineral nutrients includes maintenance of certain physicochemical processes which are essential to life [61]. Minerals such as iron, zinc, aluminium, copper, and manganese help in the prevention of some malnutrition incidences [62]. Essential minerals are of two groups: major minerals which are needed in amounts greater than 100 mg per day and constitute about 1% of the body weight and trace minerals required in less than 100 mg per day and constituting less than 0.01% of body weight [63]. Calcium is responsible for bone and muscle formation and maintenance and prevents osteopenia and osteoporosis caused by some chemotherapeutic agents in the body [64]. Furthermore, calcium helps in clotting of blood, muscle contraction, and synaptic transmission of nerve impulses [65]. Up to 1200 mg is needed in daily diets [66]. Taking into consideration the recommendations of NRC [67] and Turan et al. [67], the lowest calcium content (1590 mg) reported in the present trial has the potential to supply more than the RDA of 1200 mg in humans. Regular consumption of* S. nigrum *could be used as a source of magnesium for the prevention of ischemic heart disease which is result of this mineral's deficiency [68].

The Na/K ratio in the body is linked with the prevention of hypertension and values greater than one are thought to increase chances of hypertension [25, 69, 70]. The values in* S. nigrum *samples on all soil types in this study are less than 0.6 indicating the potential of this wild vegetable in providing a healthy and balanced diet. Magnesium is reported to be efficient in the metabolism of carbohydrates and lipids, involved in cellular respiration, and also useful in general cellular biochemistry and functions [71].

### 5.1. Effect of Soil Types on Mineral Uptake and Accumulation in* S. nigrum*

Generally in this study, all* S. nigrum *samples from different soil types experienced significant variations in their mineral contents. However, the quantities observed in all plants from different soil treatments were higher than those reported in many other studies but on the same plant [44, 72]. All minerals both essential and nonessential accumulated in abundance in the plant regardless of soil types. However, comparing with the recommended levels of ADA, NHMRC, and FDA [73–75], all mineral contents in* S. nigrum *were within the RDA levels. Minerals maintain certain physicochemical processes in the human body which are essential to life [61]. Moreover, in plants, deficiency problems are always as a result of lack of some essential elements. Micronutrients such as iron, zinc, aluminium copper, and manganese accumulated in* S. nigrum *can play a role in the prevention of malnutrition [62]. Malnutrition as a result of micronutrient deficiencies has been reported to claim more than 3.7 million in children per year [76]. Zinc helps in protein synthesis, normal body development, and recovery from illness [77]. Its deficiency is associated with poor wound healing, growth retardation, loss of appetite, and loss of taste.

However, their excess intake by plants become toxic and poses a hazard to consumer health [19]. Some food plants can tolerate heavy metal uptake up to 10,000 mg/kg including* S. nigrum *[78]. These trace elements are said to be toxic even at low concentrations [14]. However, high vitamins present in the same food component alleviate their toxicity. It is necessary to check the safety levels of the food source such as vegetables which are meant for human consumption. According to the reports of Cui et al. and Li et al. [42, 43] translocation factor (TF) given as [metals] shoot/[metals] root is a very good indicator to ascertain the hyperaccumulating capacity of a plant. Ability of the plant root to translocate higher quantities of heavy metals to the shoot parts increases the shoot/root ratio > 1; the higher the metal value than 1, the higher the hyperaccumulating capacity of the plant.

The present minerals reported in the current work, including iron, aluminium, zinc, manganese, and copper, indicate that* S. nigrum *is a potential reservoir of these minerals. In the current study, soil types significantly affected mineral compositions in* S. nigrum. *The consumption of vegetables can supply important minerals in abundance and each enhances one activity or the other in human physiology and helps prevent many chronic diseases [46]. Accumulation of micronutrients was observed to be the highest at the flowering stage and this was between the 4th and 5th weeks after transplanting [78]. However, none of the microelements was accumulated at higher levels to become toxic in the plant. Low levels of accumulated trace metals in all soil texture types in this study may be due to their low amount in the soils and no source substance of these metals was applied to the plant under cultivation.

## 6. Conclusion

The current study established that soil types have effect on mineral nutrients and antinutrients accumulation in* S. nigrum. *Accumulation of high mineral nutrients in* S. nigrum* shoot reflects a characteristic of a nutrient supplying vegetable. High fibre contents confirm its digestive effectiveness in humans. Also, the high protein content affirmed that the plant is worth of an immune booster. Plants cultivated on clay loam, silty clay loam, and loam soils accumulated elevated nutritional compositions and abundant antinutrients such as phytate, oxalate, and alkaloids. However, sandy clay loam soils accumulated the highest saponins. The best recommended periods to harvest the plant for nutritional purpose were between the 4th and 5th weeks after transplanting. Tangible values of vitamins A, C, and E recorded in this study added to the antioxidant properties of the plant. Macro- and micronutrients in* S. nigrum* in this study are within the RDA for consumption. Based on this plant's nutritional compositions,* Solanum nigrum *should be domesticated and this plant responds very well to clay loam and silty clay loam soils.

## Figures and Tables

**Table 1:** Physical characteristics of formulated experimental soil types.

Soil types	Sand particles%	Silt particles%	Clay particles%
Control (ST_0_)	60	30	10
Sandy clay loam (ST_1_)	66	13	21
Silty clay loam (ST_2_)	10	60	30
Clay loam (ST_3_)	36	30	34
Loam (ST_4_)	40	40	20

**Table 2:** Nutrient composition and physical properties of formulated experimental soils for trials 1 and 2.

Soil types	ST_0_	ST_1_	ST_2_	ST_3_	ST_4_	ST_0_	ST_1_	ST_2_	ST_3_	ST_4_
Soil mineral composition (mg/kg)	First trial					Second trial				
P	68 ± 1^b^	84 ± 0^a^	62 ± 1^c^	60 ± 0^cd^	63 ± 1^c^	74 ± 0^b^	73 ± 0^a^	67 ± 0^c^	68 ± 0^cd^	72 ± 0^c^
k	524 ± 0^a^	467 ± 1^d^	524 ± 0^a^	519 ± 1^b^	482 ± 0^c^	582 ± 0^a^	549 ± 0^d^	562 ± 0^a^	549 ± 0.5^b^	543 ± 0^c^
N	3.4 ± 1^c^	3.6 ± 0^c^	5 ± 0.5^a^	4.8 ± 0^b^	4.6 ± 0^b^	3.8 ± 0.4^c^	4.0 ± 0^c^	5 ± 0.5^a^	4.9 ± 0^b^	4.8 ± 0^b^
Ca	1389 ± 0.5^a^	1357 ± 0.5^b^	1278 ± 0.5^e^	1318 ± 1.0^c^	1290 ± 0.5^d^	1434 ± 0.5^a^	1399 ± 0.5^b^	1323 ± 0.5^e^	1360 ± 0.5^c^	1393 ± 0.5^d^
Mg	332 ± 0.1^b^	347 ± 0.5^a^	316 ± 0.0^d^	321 ± 0.0^c^	330 ± 0.5^b^	350 ± 0.1^b^	336 ± 0.5^a^	328 ± 0.0^d^	325 ± 0.0^c^	339 ± 0.5^b^
Zn	6.0 ± 0.3^b^	8.1 ± 0.5^a^	5.9 ± 0.1^bc^	5.3 ± 0.1^c^	6.2 ± 0^b^	3.9 ± 0.3^b^	4.7 ± 0.5^a^	8.4 ± 0.1^bc^	4.5 ± 0.5^c^	5.3 ± 0^b^
Mn	29 ± 1^d^	66 ± 0^a^	45 ± 0^b^	38 ± 0^c^	45 ± 0^b^	57 ± 0.1^d^	80 ± 0^a^	73 ± 0^b^	77 ± 0^c^	75 ± 0^b^
Cu	10.5 ± 0^c^	15.6 ± 0^a^	10.6 ± 1^c^	10.3 ± 0^c^	11.4 ± 0^b^	8.8 ± 0^c^	7.4 ± 0^a^	10.±0.5^c^	7.8 ± 0^c^	8.0 ± 0^b^
Organic content (%)	4 ± 0.5^c^	3.9 ± 0^c^	5 ± 1^a^	4.5 ± 0^b^	4.8 ± 1^a^	4.2 ± 0.5^c^	4.4 ± 0^c^	4.9 ± 0.7^a^	4.7 ± 0^b^	4.7 ± 0.7^b^
pH	6.22 ± 0^a^	5.7 ± 1^b^	5.7 ± 0^b^	5.63 ± 1^b^	5.63 ± 1^b^	6.32 ± 0^a^	5.54 ± 0^b^	6.70 ± 0^b^	6.50 ± 0^b^	6.51 ± 0^b^
Clay%	10 ± 1^c^	21 ± 1^b^	30 ± 0^a^	34 ± 0^a^	20 ± 1^b^	10 ± 0^c^	21 ± 0^b^	30 ± 0^a^	34 ± 0^a^	20 ± 1^b^
Sand%	60 ± 1^a^	66 ± 0.5^a^	10 ± 0.7^c^	36 ± 0^c^	40 ± 0.5^b^	60 ± 0.5^a^	66 ± 0.5^a^	10 ± 0.5^c^	36 ± 0^c^	40 ± 0.5^b^
Silt%	30 ± 1^c^	13 ± 0^d^	60 ± 0^a^	30 ± 0.1^c^	40 ± 0^b^	30 ± 0.7^c^	13 ± 0^d^	60 ± 0^a^	30 ± 0.1^c^	40 ± 0^b^

Values shown are means ± standard deviation; different letters down a column represent significant differences at *p* < 0.01.

**Table:** (a) Effect of soil types on *S. nigrum *proximate composition at 4 weeks in the first and second trials.

Soil types	Crude fibre%		Ash content%		Moisture content%		Protein%	
	1st trial	2nd trial	1st trial	2nd trial	1st trial	2nd trial	1st trial	2nd trial
ST0	19.24 ± 0.03^ab^	18.71 ± 0.02^b^	11.25 ± 0.02^b^	11.18 ± 0.02^ab^	94.48 ± 0.01^c^	93.50 ± 0.01^c^	36.72 ± 0.02^b^	34.47 ± 0.03^c^
ST1	19.44 ± 0.02^ab^	18.95 ± 0.02^b^	11.46 ± 0.01^b^	11.14 ± 0.02^ab^	95.97 ± 0.02^ab^	93.59 ± 0.02^c^	36.89 ± 0.01^b^	34. 53 ± 0.01^c^
ST2	19.83 ± 0.01^a^	19.07 ± 0.03^a^	12.10 ± 0.03^a^	11.54 ± 0.01^a^	96.05 ± 0.03^a^	94.64 ± 0.02^a^	38.90 ± 0.02^a^	36.23 ± 0.02^b^
ST3	19.95 ± 0.01^a^	19.35 ± 0.01^a^	12.42 ± 0.02^a^	11.27 ± 0.03^ab^	95.98 ± 0.02^ab^	93.91 ± 0.03^b^	38.98 ± 0.01^a^	36.89 ± 0.01^a^
ST4	19.56 ± 0.02^a^	19.17 ± 0.03^a^	12.25 ± 0.02^a^	11.18 ± 0.02^ab^	95.40 ± 0.02^b^	93.84 ± 0.02^b^	38.34 ± 0.01^a^	37.00 ± 0.01^a^

**Table:** (b) Effect of soil types on *S. nigrum *proximate composition at 4 weeks in the first and second trials

Soil types	Lipid%		Carbohydrate%		Energy value (kcal/100 g)	
	1st trial	2nd trial	1st trial	2nd trial	1st trial	2nd trial
ST0	4.86 ± 0.03^a^	4.42 ± 0.01^a^	15.34 ± 0.06^b^	17.99 ± 0.02^a^	180.49 ± 0.01^c^	172.51 ± 0.01^d^
ST1	4.35 ± 0.02^ab^	4.20 ± 0.03^a^	16.85 ± 0.02^a^	17.83 ± 0.01^a^	200.47 ± 0.02^b^	193.38 ± 0.01^c^
ST2	3.77 ± 0.01^b^	3.70 ± 0.06^b^	13.59 ± 0.03^c^	15.16 ± 0.02^b^	203.56 ± 0.03^a^	194.00 ± 0.03^b^
ST3	3.57 ± 0.02^c^	3.46 ± 0.01^b^	12.74 ± 0.02^d^	14.17 ± 0.02^c^	203.44 ± 0.02^a^	195.89 ± 0.02^a^
ST4	3.58 ± 0.02^c^	3.53 ± 0.03^b^	12.81 ± 0.01^d^	14.23 ± 0.02^c^	205.79 ± 0.03^a^	195.03 ± 0.02^ab^

**Table:** (c) Effect of soil types on in *S. nigrum *vitamins at 4 weeks in the first and second trials

Soil types	Vitamin A (mg/kg)		Vitamin C (mg/kg)		Vitamin E (mg/kg)	
	1st trial	2nd trial	1st trial	2nd trial	1st trial	2nd trial
ST0	117 ± 0.07^b^	108 ± 0.01^c^	100 ± 0.06^b^	96 ± 0.02^b^	250 ± 0.01^c^	238 ± 0.01^c^
ST1	125 ± 0.02^b^	116 ± 0.05^c^	104 ± 0.02^b^	99 ± 0.01^b^	257 ± 0.02^b^	227 ± 0.01^d^
ST2	129 ± 0.01^b^	120 ± 0.06^b^	111 ± 0.03^a^	101 ± 0.02^a^	262 ± 0.03^a^	240 ± 0.03^c^
ST3	133 ± 0.02^a^	123 ± 0.01^a^	114 ± 0.02^a^	108 ± 0.02^a^	264 ± 0.02^a^	253 ± 0.02^b^
ST4	137 ± 0.02^a^	128 ± 0.03^a^	118 ± 0.01^a^	108 ± 0.02^a^	269 ± 0.03^a^	261 ± 0.02^a^

Different letters down the same column represent significant difference at *p* < 0.01.

**Table 4:** Effect of soil types on *S. nigrum* antinutrient composition.

Soil types	Phytate content%		Oxalate content%		Saponin content%		Alkaloid content%	
	1st trial	2nd trial	1st trial	2nd trial	1st trial	2nd trial	1st trial	2nd trial
ST0	0.84 ± 0.02^d^	0.88 ± 0.03^d^	11.00 ± 0.03^e^	11.20 ± 0.03^c^	96.00 ± 0.02^b^	97.80 ± 0.03^a^	43.03 ± 0.02^c^	42.08 ± 0.03^c^
ST1	1.06 ± 0.06^b^	1.00 ± 0.02^b^	12.47 ± 0.06^d^	12.11 ± 0.02^b^	97.00 ± 0.03^a^	96.90 ± 0.01^b^	43.22 ± 0.01^c^	43.14 ± 0.02^b^
ST2	1.05 ± 0.02^b^	0.96 ± 0.03^b^	13.68 ± 0.02^c^	12.67 ± 0.01^b^	95.33 ± 0.02^c^	95.17 ± 0.01^c^	45.76 ± 0.02^a^	43.98 ± 0.02^a^
ST3	1.17 ± 0.03^a^	1.15 ± 0.01^a^	15.25 ± 0.01^a^	14.60 ± 0.01^a^	94.10 ± 0.01^d^	94.17 ± 0.02^d^	45.66 ± 0.03^a^	44.10 ± 0.01^a^
ST4	0.90 ± 0.01^c^	0.98 ± 0.01^c^	14.67 ± 0.02^b^	13.77 ± 0.03^ab^	96.00 ± 0.01^b^	96.57 ± 0.03^b^	44.29 ± 0.01^b^	43.84 ± 0.01^a^

Values shown are mean ± SD. Different letters down the same column represent significant difference at *p* < 0.01.

**Table:** (a) Effect of soil types on nitrogen and phosphorus accumulation in *S. nigrum* during the first and second trials.

Soil types	Nitrogen composition%							Phosphorus composition%						
	Plant age after transplanting (week)													
	0	1	2	3	4	5	6	0	1	2	3	4	5	6
(i) The first trial														
ST_0_	2.12	4.56 ± 0.02^d^	5.58 ± 0.02^c^	6.12 ± 0.01^c^	6.32 ± 0.03^c^	6.40 ± 0.03^c^	6.42 ± 0.02^c^	0.20	0.40 ± 0.01^a^	0.63 ± 0.0^a^	0.68 ± 0.01^c^	0.65 ± 0.01^b^	0.69 ± 0.01^c^	0.67 ± 0.03^b^
ST_1_	2.12	4.63 ± 0.01^c^	5.80 ± 0.03^b^	6.24 ± 0.03^b^	6.40 ± 0.02^c^	6.47 ± 0.02^b^	6.46 ± 0.03^c^	0.20	0.43 ± 0.02^a^	0.66 ± 0.02^a^	0.70 ± 0.02^c^	0.70 ± 0.02^b^	0.73 ± 0.02^b^	0.72 ± 0.01^b^
ST_2_	2.12	5.67 ± 0.01^a^	6.09 ± 0.02^a^	6.26 ± 0.02^b^	6.51 ± 0.01^b^	6.58 ± 0.03^b^	6.55 ± 0.02^b^	0.20	0.46 ± 0.02^a^	0.68 ± 0.01^a^	0.82 ± 0.02^a^	0.85 ± 0.01^a^	0.89 ± 0.03^a^	0.90 ± 0.03^a^
ST_3_	2.12	5.52 ± 0.02^a^	6.18 ± 0.02^a^	6.43 ± 0.01^a^	6.75 ± 0.01^a^	6.83 ± 0.02^a^	6.83 ± 0.01^a^	0.20	0.45 ± 0.01^a^	0.69 ± 0.01^a^	0.75 ± 0.01^b^	0.89 ± 0.02^a^	0.93 ± 0.02^a^	0.92 ± 0.01^a^
ST_4_	2.12	4.96 ± 0.02^b^	5.83 ± 0.01^b^	6.20 ± 0.01^b^	6.68 ± 0.03^a^	6.76 ± 0.02^a^	6.74 ± 0.02^a^	0.20	0.44 ± 0.02^a^	0.68 ± 0.02^a^	0.73 ± 0.02^b^	0.80 ± 0.02^a^	0.86 ± 0.01^a^	0.85 ± 0.02^a^

(ii) The second trial														
ST_0_	2.13	4.59 ± 0.02^d^	5.38 ± 0.02^d^	5.82 ± 0.02^c^	5.88 ± 0.02^c^	5.91 ± 0.03^d^	5.90 ± 0.03^d^	0.25	0.44 ± 0.02^b^	0.58 ± 0.03^b^	0.63 ± 0.02^b^	0.60 ± 0.02^c^	0.63 ± 0.03^b^	0.61 ± 0.01^b^
ST_1_	2.13	5.03 ± 0.01^d^	5.43 ± 0.03^c^	5.94 ± 0.01^b^	6.04 ± 0.02^c^	6.06 ± 0.02^c^	6.04 ± 0.02^c^	0.25	0.47 ± 0.02^b^	0.62 ± 0.03^a^	0.67 ± 0.02^b^	0.65 ± 0.02^b^	0.66 ± 0.01^b^	0.62 ± 0.02^b^
ST_2_	2.13	5.42 ± 0.01^b^	5.59 ± 0.03^b^	6.00 ± 0.01^a^	6.21 ± 0.02^c^	6.20 ± 0.02^b^	6.22 ± 0.02^b^	0.25	0.50 ± 0.02^a^	0.64 ± 0.02^a^	0.74 ± 0.02^a^	0.73 ± 0.01^a^	0.75 ± 0.01^a^	0.74 ± 0.01^a^
ST_3_	2.13	5.62 ± 0.02^a^	5.98 ± 0.01^a^	6.13 ± 0.01^a^	6.35 ± 0.01^a^	6.38 ± 0.02^a^	6.33 ± 0.01^a^	0.25	0.55 ± 0.03^a^	0.67 ± 0.01^a^	0.69 ± 0.01^b^	0.77 ± 0.02^a^	0.79 ± 0.01^a^	0.80 ± 0.01^a^
ST_4_	2.13	5.16 ± 0.01^c^	5.51 ± 0.02^b^	6.10 ± 0.02^a^	6.18 ± 0.01^b^	6.22 ± 0.01^b^	6.23 ± 0.03^b^	0.25	0.54 ± 0.01^a^	0.65 ± 0.03^a^	0.68 ± 0.01^b^	0.71 ± 0.02^a^	0.71 ± 0.02^a^	0.69 ± 0.02^b^

Values shown are mean ± SD. Different letters down the same column represent significant difference at *p* < 0.01.

**Table:** (b) Effect of soil types on *S. nigrum *calcium and potassium composition in the first trial and the second trial.

Soil types	Calcium composition (mg/kg)							Potassium composition (mg/kg)						
	Plant age after transplanting (week)													
	0	1	2	3	4	5	6	0	1	2	3	4	5	6
(i) The first trial														
ST_0_	450	0890 ± 0.01^c^	1220 ± 0.02^b^	1410 ± 0.01^c^	1670 ± 0.01^c^	1750 ± 0.02^c^	1740 ± 0.01^b^	1970	2870 ± 0.01^c^	5760 ± 0.03^c^	6160 ± 0.03^c^	6460 ± 0.01^e^	6510 ± 0.02^d^	6490 ± 0.03^d^
ST_1_	450	0940 ± 0.02^b^	1240 ± 0.01^b^	1510 ± 0.03^b^	1509 ± 0.02^c^	1680 ± 0.01^d^	1620 ± 0.02^c^	1970	2890 ± 0.00^c^	5960 ± 0.02^b^	6270 ± 0.01^c^	7010 ± 0.01^c^	7100 ± 0.03^c^	7100 ± 0.02^c^
ST_2_	450	1110 ± 0.03^a^	1280 ± 0.01^a^	1550 ± 0.03^b^	1600 ± 0.03^c^	1660 ± 0.01^d^	1620 ± 0.02	1970	3110 ± 0.01^a^	6600 ± 0.01^a^	6580 ± 0.01^b^	7190 ± 0.02^c^	7110 ± 0.02^c^	7080 ± 0.01^c^
ST_3_	450	1100 ± 0.01^a^	1240 ± 0.03^b^	1640 ± 0.01^a^	2000 ± 0.01^a^	2100 ± 0.03^a^	1940 ± 0.01^a^	1970	2940 ± 0.01^b^	6570 ± 0.01^a^	7400 ± 0.03^a^	8700 ± 0.03^a^	8760 ± 0.01^a^	8770 ± 0.01^a^
ST_4_	450	0940 ± 0.01^b^	1240 ± 0.01^b^	1450 ± 0.01^c^	1880 ± 0.01^b^	1970 ± 0.01^b^	1900 ± 0.01^a^	1970	2880 ± 0.02^c^	5750 ± 0.01^c^	7280 ± 0.02^a^	7750 ± 0.01^b^	7820 ± 0.01^b^	7810 ± 0.02^b^

(ii) The second trial														
ST_0_	460	0900 ± 0.03^b^	1200 ± 0.01^a^	1370 ± 0.02^d^	1580 ± 0.01^b^	1650 ± 0.03^c^	1630 ± 0.02^c^	2100	2970 ± 0.03^b^	5380 ± 0.01^c^	5460 ± 0.02^d^	5960 ± 0.02^d^	5990 ± 0.03^d^	6000 ± 0.02^d^
ST_1_	460	0980 ± 0.02^b^	1220 ± 0.0^a^	1400 ± 0.03^c^	1510 ± 0.02^b^	1580 ± 0.03^c^	1590 ± 0.03^c^	2100	2990 ± 0.02^b^	5760 ± 0.03^b^	5970 ± 0.01^c^	6010 ± 0.03^d^	6040 ± 0.03^d^	6020 ± 0.02^d^
ST_2_	460	1100 ± 0.03^a^	1250 ± 0.03^a^	1510 ± 0.03^b^	1580 ± 0.01^b^	1660 ± 0.02^c^	1650 ± 0.01^c^	2100	3190 ± 0.02^a^	5960 ± 0.02^a^	5980 ± 0.02^c^	7190 ± 0.02^c^	7240 ± 0.03^c^	7230 ± 0.02^c^
ST_3_	460	1100 ± 0.02^a^	1230 ± 0.0^a^	1610 ± 0.03^a^	1710 ± 0.01^a^	1820 ± 0.01^a^	1840 ± 0.01^a^	2100	3080 ± 0.02^a^	6040 ± 0.01^a^	6800 ± 0.01^a^	8700 ± 0.01^a^	8750 ± 0.01^a^	8760 ± 0.01^a^
ST_4_	460	0960 ± 0.01^b^	1220 ± 0.0^a^	1420 ± 0.02^c^	1620 ± 0.02^a^	1710 ± 0.01^b^	1720 ± 0.01^b^	2100	2980 ± 0.01^b^	5450 ± 0.01^c^	6480 ± 0.01^b^	7750 ± 0.01^b^	8100 ± 0.0^b^	8060 ± 0.01^b^

Values shown are mean ± SD. Different letters down the same column represent significant difference at *p* < 0.01.

**Table:** (c) Effect of soil types on *S. nigrum* sodium and magnesium composition in the first trial and the second trial.

Soil types	Sodium composition (mg/kg)							Magnesium composition (mg/kg)						
	Plant age after transplanting (week)													
	0	1	2	3	4	5	6	0	1	2	3	4	5	6
(i) The first trial														
ST0	85	450 ± 0.02^b^	500 ± 0.03^e^	740 ± 0.02^d^	862 ± 0.03^d^	870 ± 0.03^e^	930 ± 0.03^d^	1200	1900 ± 0.01^b^	4100 ± 0.02^b^	4610 ± 0.02^b^	5225 ± 0.01^b^	5698 ± 0.01^b^	5360 ± 0.03^b^
ST1	85	440 ± 0.01^b^	600 ± 0.02^d^	800 ± 0.01^c^	860 ± 0.02^c^	876 ± 0.01^d^	922 ± 0.03^d^	1200	2100 ± 0.03^a^	4900 ± 0.03^b^	4800 ± 0.01^b^	5610 ± 0.02^b^	0.6086 ± 0.02^a^	5910 ± 0.01^b^
ST2	85	514 ± 0.03^a^	600 ± 0.02^c^	800 ± 0.03^b^	910 ± 0.02^b^	934 ± 0.02^c^	975 ± 0.02^c^	1200	2300 ± 0.02^a^	5100 ± 0.03^a^	5500 ± 0.02^a^	5900 ± 0.02^b^	0.6255 ± 0.02^a^	6045 ± 0.02^a^
ST3	85	500 ± 0.02^a^	600 ± 0.01^a^	800 ± 0.02^a^	912 ± 0.02^a^	930 ± 0.01^a^	976 ± 0.01^a^	1200	2500 ± 0.02^a^	5100 ± 0.02^a^	5900 ± 0.01^a^	6100 ± 0.01^a^	0.6600 ± 0.01^a^	6500 ± 0.01^a^
ST4	85	438 ± 0.02^c^	600 ± 0.02^b^	745 ± 0.02^c^	920 ± 0.02^a^	940 ± 0.03^b^	980 ± 0.02^b^	1200	2600 ± 0.03^a^	4400 ± 0.01^b^	5185 ± 0.03^a^	6012 ± 0.02^a^	6480 ± 0.01^a^	6232 ± 0.01^a^

(ii) The second trial														
ST0	80	447 ± 0.02^c^	500 ± 0.03^e^	720 ± 0.03^c^	850 ± 0.02^d^	945 ± 0.02^c^	972 ± 0.03^b^	1100	1900 ± 0.03^b^	4118 ± 0.01^b^	4668 ± 0.01^b^	5272 ± 0.03^b^	5656 ± 0.02^b^	5348 ± 0.03^b^
ST1	80	440 ± 0.03^c^	488 ± 0.03^d^	800 ± 0.02^b^	850 ± 0.02^c^	950 ± 0.03^b^	964 ± 0.02^a^	1100	2100 ± 0.03^a^	4900 ± 0.02^b^	4818 ± 0.02^b^	5613 ± 0.02^b^	6040 ± 0.03^b^	5955 ± 0.01^b^
ST2	80	520 ± 0.02^a^	610 ± 0.01^c^	800 ± 0.02^b^	934 ± 0.01^b^	938 ± 0.01^a^	958 ± 0.02^a^	1100	3000 ± 0.01^a^	5900 ± 0.01^a^	5520 ± 0.03^a^	5900 ± 0.01^b^	6224 ± 0.01^a^	6039 ± 0.02^a^
ST3	80	500 ± 0.01^b^	614 ± 0.02^a^	800 ± 0.00^a^	938 ± 0.02^a^	950 ± 0.01^a^	967 ± 0.01^b^	1100	2900 ± 0.01^a^	5104 ± 0.03^a^	5900 ± 0.01^a^	7900 ± 0.01^a^	6600 ± 0.02^a^	6500 ± 0.01^a^
ST4	80	420 ± 0.02^b^	610 ± 0.02^b^	800 ± 0.00^c^	940 ± 0.01^a^	949 ± 0.02^b^	964 ± 0.01^b^	1100	2600 ± 0.01^a^	4600 ± 0.01^b^	5122 ± 0.02^a^	6000 ± 0.02^b^	6400 ± 0.01^a^	6240 ± 0.03^a^

Values shown are mean ± SD. Different letters down the same column represent significant difference at *p* < 0.01.

**Table:** (d) Effect of soil types on* S. nigrum* zinc and iron metals uptake in the first trial and the second trial.

Soil types	Zinc composition (mg/kg)							Iron composition (mg/kg)						
	Plant age after transplanting (week)													
	0	1	2	3	4	5	6	0	1	2	3	4	5	6
(i) The first trial														
ST_0_ shoot	12.00	20.00 ± 0.01^b^	28.00 ± 0.03^b^	40.00 ± 0.03^c^	70.00 ± 0.01^a^	76.00 ± 0.02^b^	70.00 ± 0.02^b^	230	643 ± 0.02^c^	1022 ± 0.01^c^	1340 ± 0.03^d^	2530 ± 0.03^d^	2553 ± 0.03^d^	2539 ± 0.01^d^
ST_1_ shoot	12.00	22.80 ± 0.01^a^	30.00 ± 0.01^b^	49.00 ± 0.03^b^	77.00 ± 0.03^a^	81.00.±0.03^a^	80.00 ± 0.01^a^	230	782 ± 0.02^b^	1026 ± 0.03^c^	1877 ± 0.03^c^	2777 ± 0.01^c^	2739 ± 0.03^c^	2736 ± 0.03^c^
ST_2_ shoot	12.00	28.70 ± 0.03^a^	38.00 ± 0.02^a^	62.00 ± 0.03^a^	79.00 ± 0.03^a^	84.00 ± 0.03^a^	80.00 ± 0.02^a^	230	822 ± 0.03^a^	1634 ± 0.03^a^	2343 ± 0.02^b^	3084 ± 0.02^b^	3093 ± 0.01^b^	3088 ± 0.03^b^
ST_3_ shoot	12.00	29.00 ± 0.02^a^	35.00 ± 0.01^a^	58.00 ± 0.01^b^	78.00 ± 0.01^a^	84.00 ± 0.01^a^	80.00 ± 0.01^a^	230	830 ± 0.02^a^	1350 ± 0.03^b^	2669 ± 0.01^a^	3520 ± 0.03^a^	3534 ± 0.03^a^	3528 ± 0.01^a^
ST_4_ shoot	12.00	23.00 ± 0.01^a^	31.00 ± 0.02^a^	53.00 ± 0.02^b^	75.00 ± 0.02^a^	80.00 ± 0.03^a^	78.00 ± 0.02^a^	230	784 ± 0.02^b^	1106 ± 0.03^c^	2027 ± 0.01^b^	2833 ± 0.03^c^	2842 ± 0.01^c^	2844 ± 0.03^c^

(ii) The second trial														
ST_0_ shoot	14.00	22.00 ± 0.03^b^	27.00 ± 0.02^b^	36.20 ± 0.01^c^	65.00 ± 0.02^b^	70.00 ± 0.1^a^	68.00 ± 0.02^b^	230	649 ± 0.01^c^	1000 ± 0.02^b^	1270 ± 0.02^b^	2338 ± 0.01^d^	2348 ± 0.01^d^	2334 ± 0.02^d^
ST_1_ shoot	14.00	23.00 ± 0.01^b^	30.00 ± 0.02^a^	46.00 ± 0.03^b^	72.00 ± 0.02^a^	77.00 ± 0.2^a^	70.00 ± 0.01^a^	230	785 ± 0.01^b^	1011 ± 0.01^b^	1577 ± 0.02^c^	2568 ± 0.02^c^	2576 ± 0.03^c^	2563 ± 0.01^c^
ST_2_ shoot	14.00	29.20 ± 0.02^a^	35.80 ± 0.02^a^	59.00 ± 0.01^a^	72.00 ± 0.03^a^	76.00 ± 0.01^a^	72.00 ± 0.02^a^	230	828 ± 0.01^a^	1604 ± 0.01^a^	2258 ± 0.02^a^	3084 ± 0.02^b^	3092 ± 0.02^b^	3056 ± 0.02^b^
ST_3_ shoot	14.00	32.00 ± 0.01^a^	38.00 ± 0.01^a^	55.10 ± 0.02^a^	71.00 ± 0.01^a^	76.00 ± 0.01^a^	730 ± 0.01^a^	230	836 ± 0.03^a^	1290 ± 0.02^b^	2489 ± 0.01^a^	3520 ± 0.02^a^	3530 ± 0.03^a^	3522 ± 0.03^a^
ST_4_ shoot	14.00	25.60 ± 0.01^b^	28.60 ± 0.02^b^	50.50 ± 0.01^a^	70.00 ± 0.03^a^	75.00 ± 0.01^a^	710 ± 0.02^a^	230	790 ± 0.03^b^	1096 ± 0.02^b^	1986 ± 0.02^b^	2689 ± 0.02^c^	2691 ± 0.02^c^	2678 ± 0.01^c^

Values shown are mean ± SD. Different letters down the same column represent significant difference at *p* < 0.01.

**Table:** (e) Effect of soil types on *S. nigrum *copper and manganese metals uptake in the first trial and the second trial.

Soil types	Copper composition (mg/kg)						Manganese composition (mg/kg)							
	Plant age after transplanting (week)													
	0	1	2	3	4	5	6	0	1	2	3	4	5	6
(i) The first trial														
ST_0_ shoot	10.1	14.0 ± 0.01^a^	25.4 ± 0.03^a^	30.5 ± 0.02^a^	36.5 ± 0.01^a^	38.9 ± 0.02^a^	35.6 ± 0.02^a^	0.09	120 ± 0.02^a^	120 ± 0.03^c^	156 ± 0.03^d^	226 ± 0.02^b^	230 ± 0.01^c^	228 ± 0.03^b^
ST_1_ shoot	10.1	142 ± 0.01^a^	27.4 ± 0.03^a^	31.8 ± 0.03^a^	34.7 ± 0.01^a^	36.8 ± 0.01^a^	35.2 ± 0.03^a^	0.09	120 ± 0.03^a^	140 ± 0.02^b^	170 ± 0.01^c^	235 ± 0.01^a^	242 ± 0.03^a^	230 ± 0.03^a^
ST_2_ shoot	10.1	15.4 ± 0.02^a^	29.3 ± 0.01^a^	33.4 ± 0.01^a^	38.5 ± 0.03^a^	40.2 ± 0.02^a^	38.2 ± 0.01^a^	0.09	120 ± 0.03^a^	130 ± 0.01^a^	192 ± 0.03^c^	243 ± 0.01^a^	239 ± 0.01^b^	229 ± 0.01^b^
ST_3_ shoot	10.1	15.3 ± 0.02^a^	29.8 ± 0.01^a^	30.4 ± 0.01^b^	31.0 ± 0.02^b^	33.1 ± 0.01^b^	30.9 ± 0.03^b^	0.09	120 ± 0.01^a^	168 ± 0.02^a^	236 ± 0.03^a^	240 ± 0.02^a^	236 ± 0.02^b^	232 ± 0.02^a^
ST_4_ shoot	10.1	14.1 ± 0.02^a^	28.5 ± 0.01^a^	30.3 ± 0.02^b^	33.9 ± 0.01^b^	35.8 ± 0.03^a^	31.2 ± 0.03^b^	0.09	120 ± 0.03^a^	169 ± 0.03^b^	175 ± 0.02^b^	230 ± 0.01^a^	236 ± 0.02^b^	229 ± 0.01

(ii) The second trial														
ST_0_ shoot	10.8	15.4 ± 0.03^a^	23.8 ± 0.02^b^	27.8 ± 0.01^a^	30.6 ± 0.02^a^	32.8 ± 0.01^b^	32.0 ± 0.02^a^	0.1	100 ± 0.01^b^	120 ± 0.03^d^	147 ± 0.02^b^	218 ± 0.02^b^	222 ± 0.02^b^	219 ± 0.03^b^
ST_1_ shoot	10.8	15.0 ± 0.01^a^	26.6 ± 0.01^a^	28.8 ± 0.01^a^	31.7 ± 0.03^a^	33.1 ± 0.02^b^	30 ± 0.01^b^	0.1	120 ± 0.01^a^	130 ± 0.01^c^	162 ± 0.02^b^	224 ± 0.02^a^	229 ± 0.01^b^	220 ± 0.02^b^
ST_2_ shoot	10.8	16.1 ± 0.01^a^	26.8 ± 0.02^a^	30.4 ± 0.02^a^	33.6 ± 0.01^b^	35.8 ± 0.02^a^	32.0 ± 0.02^a^	0.1	120 ± 0.01^a^	120 ± 0.02^d^	181 ± 0.02^a^	226 ± 0.03^a^	230 ± 0.02^b^	226 ± 0.02^a^
ST_3_ shoot	10.8	16.3 ± 0.02^a^	27.0 ± 0.02^a^	29.9 ± 0.01^a^	30.4 ± 0.02^b^	34.1 ± 0.03^b^	31.9 ± 0.01^a^	0.1	120 ± 0.03^a^	180 ± 0.02^a^	220 ± 0.02^a^	230 ± 0.02^a^	241 ± 0.01^a^	238 ± 0.03^a^
ST_4_ shoot	10.8	15.3 ± 0.02^a^	25.6 ± 0.02^a^	28.6 ± 0.01^a^	30.4 ± 0.02^b^	33.0 ± 0.02^b^	30 ± 0.01^b^	0.1	120 ± 0.02^a^	150 ± 0.02^b^	160 ± 0.02^b^	221 ± 0.02^a^	232 ± 0.03^a^	220 ± 0.02^b^

Values shown are mean ± SD. Different letters down the same column represent significant difference at *p* < 0.01.

**Table:** (f) Effect of soil types on* S. nigrum* translocation factor on accumulation of zinc, iron, and aluminium in the first trial and the second trial.

Soil types	Zinc composition %							Iron composition %							Aluminium composition %						
	Plant age after transplanting (week)																				
	0	1	2	3	4	5	6	0	1	2	3	4	5	6	0	1	2	3	4	5	6
(i) The first trial																					
ST_0_	1.33	1.85^c^	2.33^a^	1.38^c′^	1.79^b^	2.45^a^	1.97	1.77	1.70^b^	1.76^d^	4.67^a^	0.60^b^	4.78^a^	4.81^a^	1.44	2.52^a^	2.29^d^	2.71^b^	5.42^b^	5.54^c^	5.71^c^
ST_1_	1.33	1.75^d^	2.46^a^	1.63^b^	1.87^b^	2.45^a^	2.66^a^	1.77	1.99^a^	2.36^c^	3.94^a^	0.65^b^	3.92^c^	4.16^b^	1.44	2.68^a^	2.97^a^	2.54^c^	4.88^c^	5.03^d^	5.08^d^
ST_2_	1.33	1.91^b^	2.24^b^	1.82^a^	2.40^a^	2.40^b^	2.50^a^	1.77	2.05^a^	2.63^b^	3.66^b^	0.73^a^	3.73^c^	3.70^c^	1.44	2.30^b^	2.70^b^	2.75^b^	5.69^a^	5.72^b^	5.82^b^
ST_3_	1.33	2.07^a^	2.19^c^	1.61^b^	1.90^b^	2.47^a^	2.42^a^	1.77	1.97^a^	3.17^a^	3.68^b^	0.77^a^	4.08^b^	4.14^b^	1.44	2.35^b^	2.55^c^	2.81^a^	5.62^a^	5.96^a^	5.96^a^
ST_4_	1.33	2.09^a^	2.21^b^	1.60^b^	1.88^b^	2.42^a^	2.51^a^	1.77	2.15^a^	2.56^b^	4.64^a^	0.71^a^	4.85^a^	5.16^a^	1.44	2.51^a^	2.60^c^	2.61^c^	5.53^b^	5.62^b^	5.87^b^

(ii) The second trial																					
ST_0_	1.43	2.00^b^	1.64^c^	1.34^d^	1.91^a^	2.50^a^	2.72^a^	1.75	1.70^b^	2.01^b^	1.72^d^	4.50^b^	4.51^b^	4.59^b^	1.49	2.66^b^	2.06^c^	2.87^a^	6.19^a^	6.39^a^	6.57^b^
ST_1_	1.43	1.93^c^	1.76^b^	1.64^c^	1.95^a^	2.33^b^	2.33^b^	1.75	1.99^a^	2.16^b^	2.06^c^	4.24^c^	4.23^d^	4.27^c^	1.49	2.59^b^	3.03^a^	2.50^d^	4.89^d^	5.06^c^	5.07^d^
ST_2_	1.43	1.83^d^	1.57^d^	1.90^a^	1.87^b^	2.05^c^	2.05^d^	1.75	2.05^a^	2.28^a^	2.60^b^	4.45^b^	4.44^c^	4.54^b^	1.49	2.30^c^	2.55^b^	2.91^a^	5.74^b^	5.79^b^	5.92^c^
ST_3_	1.43	2.28^a^	1.84^a^	1.78^a^	1.97^a^	2.17^c^	2.28^c^	1.75	1.96^a^	2.03^b^	3.01^a^	6.41^a^	6.41^a^	6.68^a^	1.49	2.37^c^	2.55^b^	2.83^b^	5.08^c^	5.14^c^	5.24^d^
ST_4_	1.43	1.87^a^	1.51^d^	1.78^a^	1.94^a^	2.34^b^	2.44^b^	1.75	2.14^a^	1.97^c^	3.00^a^	4.60^b^	4.59^b^	4.68^b^	1.49	3.00^a^	2.53^b^	2.70^c^	6.13^a^	6.30^a^	6.75^a^

Values shown are mean ± SD. Different letters down the same column represent significant difference at *p* < 0.01.

**Table:** (g) Effect of soil types on *S. nigrum *translocation factor accumulation on copper and manganese in the first trial and the second trial.

Soil types	Copper composition %						Manganese composition %							
	Plant age after transplanting (week)													
	0	1	2	3	4	5	6	0	1	2	3	4	5	6
(i) The first trial														
ST_0_ shoot	1.36	1.71^a^	1.79^a^	1.69^c^	1.81^b^	2.05^b^	2.12^b^	1.35	1.59^a^	1.68^b^	1.02^b^	1.11^b^	1.17^a^	1.19^a^
ST_1_ shoot	1.36	1.71^a^	1.57^c^	1.70^b^	1.74^c^	2.00^b^	2.06^c^	1.35	1.58^a^	1.79^a^	1.06^b^	1.18^b^	1.16^a^	1.14^a^
ST_2_ shoot	1.36	1.71^a^	1.73^a^	1.76^b^	1.93^b^	2.1^b^	2.25^b^	1.35	1.53^a^	1.79^a^	1.18^a^	1.21^a^	1.15^a^	1.13^a^
ST_3_ shoot	1.36	1.56^b^	1.67^b^	1.75^b^	2.52^a^	3.00^a^	3.03^a^	1.35	1.57^a^	1.84^a^	1.13^a^	1.14^b^	1.13^a^	1.16^a^
ST_4_ shoot	1.36	1.76^a^	1.64^b^	2.05^a^	1.73^c^	1.79	3.00^a^	1.35	1.58^a^	1.81^a^	1.02^b^	1.15^b^	1.08^a^	1.11^a^

(ii) The second trial														
ST_0_ shoot	1.21	1.75^a^	1.75^a^	1.36^c^	1.76^b^	1.93^b^	2.08^a^	1.26	1.44^b^	1.58^b^	1.41^b^	1.13^a^	1.16^a^	1.27^a^
ST_1_ shoot	1.21	1.74^a^	1.61^b^	1.45^b^	1.58^c^	1.38^c^	1.36c	1.26	1.53^b^	1.06^d^	1.30^c^	1.14^a^	1.21^a^	1.18^b^
ST_2_ shoot	1.21	1.46^b^	1.76^a^	1.45^b^	1.54^c^	1.88^b^	1.95^b^	1.26	1.59^a^	1.14^c^	1.66^a^	1.14^a^	1.21^a^	1.21^a^
ST_3_ shoot	1.21	1.44^b^	1.60^b^	1.91^a^	1.93a	2.10^a^	2.18^a^	1.26	1.55^a^	1.86^a^	1.13^d^	1.15^a^	1.20^a^	1.24^a^
ST_4_ shoot	1.21	1.41^b^	1.57^b^	1.80^a^	1.86^a^	1.94^b^	1.99^b^	1.26	1.63^a^	1.02^d^	1.48^b^	1.13^a^	1.20^a^	1.18^b^

Values shown are mean ± SD. Different letters down the same column represent significant difference at *p* < 0.01.
